# MYO1G promoter hypomethylation correlates with its mRNA expression, lymphocyte infiltration, and immunotherapy response in melanoma

**DOI:** 10.3389/fonc.2025.1585450

**Published:** 2025-06-10

**Authors:** Yonghua Xia, Yushu Zhang, Chenxi Zheng, Junbo Wang, Wenyu Di, Mengjie Zhang, Minglei Yang

**Affiliations:** ^1^ The Department of Dermatovenereology, The First Affiliated Hospital of Xinxiang Medical University, Weihui, China; ^2^ The Department of Dermatology, Henan Second Provincial People’s Hospital, Zhengzhou, China; ^3^ Department of Orthopedics, The First Affiliated Hospital of Xinxiang Medical University, Weihui, China; ^4^ Department of Pathology, The First Affiliated Hospital of Xinxiang Medical University, Weihui, China; ^5^ Department of Pathology, The First Affiliated Hospital of Zhengzhou University, Zhengzhou, Henan, China

**Keywords:** immune cell infiltration, promoter methylation, *MYO1G*, immune checkpoint, immunotherapy

## Abstract

**Objectives:**

Myosin IG (*MYO1G*) plays a vital role in triggering an immune response via regulating T cell migration to detect antigen-presenting cells. However, the biological and clinical significance of *MYO1G* DNA methylation and gene expression in melanoma and its immune microenvironment remains unknown.

**Materials and methods:**

We investigated and corroborated the correlations of *MYO1G* DNA methylation with gene expression, and clinicopathologic parameters in 461 melanomas from The Cancer Genome Atlas (TCGA). Subsequently, we associated *MYO1G* gene expression with overall survival in two independent cohorts including 94 immunotherapy-treated melanoma patients and 54 stage IV melanoma patients, respectively. Finally, the quantitative methylation-specific PCR (qMSP) assays were developed to measure the methylation levels of the cg22111043 and cg10673833 CpG sites located on *MYO1G* promoter region, and targeted bisulfite sequencing assay was used to validate accuracy of qMSP. We linked the methylation levels of the two CpG sites with *MYO1G* expression and progression-free survival in our cohort of 104 melanoma patients treated with immunotherapy. we used the AI-based cell segmentation and classification software Hover-Net to perform cell count and statistical analysis on the whole-slide images of pathology from 104 melanoma patients.

**Results:**

We observed that *MYO1G* gene expression exhibited a significantly inverse correlation with its promoter methylation. Moreover, hypomethylation in *MYO1G* promoter (corresponding to high gene expression level) was significantly associated with enhanced infiltration levels of immune cells (CD8+ T cell, M1 macrophage, activated natural killer cells estimated by gene expression), increased cytolytic activity, augmented expression level of immune checkpoint molecules (*PDCD1*, *LAG3*, *CTLA4*, *CD274*, *BTLA*, *TIGIT*, and *HAVCR2*) and favorable prognosis in melanoma. In the independent melanoma cohorts receiving immune checkpoint blockade treatment, high *MYO1G* expression was significantly linked to improved clinical outcome. In our cohort, patients with *MYO1G* promoter hypomethylation showed significantly elevated tumor-infiltrating lymphocytes level and prolonged progression-free survival after immunotherapy.

**Conclusion:**

Our study highlights *MYO1G* promoter methylation as a key regulator of gene expression and a potential prognostic and predictive biomarker for immunotherapy response in melanoma. These findings offer new insights into the role of *MYO1G* in enhancing immune response in tumors.

## Introduction

Melanoma is a highly aggressive and fatal cancer, which accounts for up to 1.5% of all cancer-related deaths (1). Immune checkpoint blockade-based immunotherapy has provided remarkable clinical benefits to melanoma patients, but most ‘immune-cold’ melanomas without T cells infiltration in tumor microenvironment are not responsive (2). T cell-mediated immunotherapy depend on the success of T cell priming, trafficking, infiltration, as well as antigen recognition and killing of the tumor (3). The migration of immune cells to the tumor site is essential for initiating an anti-tumor immune response to eliminate tumor cells, subsequently impacting patient clinical prognosis and immunotherapy response (4, 5). Antigen-presenting cells such as dendritic cells, macrophages and B cells migrate to tumor regions to capture tumor antigens, after which these cells present the antigens to T cells and then prime T cell response (6). Immune effector cells such as activated CD8+ T cells and NK cells migrate to a tumor and eliminate cancer cells (7, 8). Immune cell migration in complex microenvironment is regulated by intracellular and extracellular factors. Chemokines guide the migration of both immunoactive and immunosuppressive cell types to tumor sites (9). In addition, actomyosin cytoskeleton plays a key role in immune cell migration by controlling the intrinsic nature of leukocytes (10).

The *MYO1G* gene is located on the short arm of chromosome 7 (11). It encodes a plasma membrane-associated class I myosin, which is involved in the regulation of the cytoskeleton and highly expressed in lymphocytes (11, 12). The unconventional myosin *MYO1G*, an actin-based motor protein, plays a crucial role in mechanotransduction and immune regulation by coordinating cytoskeletal dynamics at the immunological synapse (IS). Studies suggest that *MYO1G* modulates T cell receptor (TCR) signaling and immune synapse stability, influencing T cell activation and exhaustion (13). Its involvement in membrane remodeling and actin organization further implicates *MYO1G* in regulating immune cell motility and interactions with antigen-presenting cells (14). *MYO1G* generates membrane tension and regulates T cell migration by cell-intrinsic mechanism (14, 15). In addition, *MYO1G* plays a vital role in maintaining cell stiffness (16), adhesion, and migration of B cells (17, 18). B cell and T lymphocytes filtration to tumor are associated with immune checkpoint blockade-based treatment response (19–21). With regard to DNA methylation, the circulating tumor DNA methylation of *MYO1G* has been reported to be a promising biomarker for the diagnosis and disease monitoring of colorectal cancer (22) and Hepatocellular Carcinoma (23). In addition, the overexpression of *MYO1G* is detected in peripheral blood mononuclear cells from pediatric leukemia patients (24). MYO1G is a new potential markers of mixed lymphocyte reaction response (25). Our previous study demonstrated that *MYO1G* gene expression was negatively associated with promoter methylation in squamous cell lung carcinoma (26). However, how DNA methylation of *MYO1G* impacts its gene expression, tumor immune microenvironment, and clinical outcome of immunotherapy for melanoma patients remains unknown.

DNA methylation is an important epigenetic modification regulating gene expression. Alterations in DNA methylation frequently occur in various tumor types, impacting lymphocytes infiltration and immunotherapy response (27). It remains unknown that the effect of *MYO1G* DNA methylation on the gene expression, immune cell infiltration and immunotherapy response of melanoma. In this study, we investigate the associations of *MYO1G* DNA methylation with gene expression, immune cell infiltration and clinicopathological features, as *MYO1G* is highly expressed in lymphocytes and regulates lymphocyte migration. Our data shows that *MYO1G* promoter DNA methylation might regulate its gene expression and immune cell infiltration, therefore affecting clinical outcome.

## Material and methods

### Sample collection and resources

The analyzed results in this study are partly based on skin cutaneous tumor (SKCM) data generated by The Cancer Genome Atlas Research Network (TCGA, http://cancergenome.nih.gov/). The methylation, RNA-seq and clinicopathological data of 472 skin cutaneous tumor samples were collected from TCGA. Out of these samples, 461 samples included clinical information. Therefore, 358 metastatic SKCM samples were used as a discovering dataset; 103 primary SKCM samples as a validating dataset. Expression profiles generated by Illumina human-6 v2.0 expression beadchip platform and clinical data of 54 stage IV melanomas were required from Gene Expression Omnibus database with the accession number of GSE22153 (28).RNA-seq data of tumor tissues derived from 91 melanoma patients treated with immunotherapy were obtained from the European Nucleotide Archive by accession number PRJEB23709, and the clinical summary of patients was available in **Supplementary Table S2** of the original paper (29). The transcriptome and clinical data of 53 baseline tumors from a cohort of melanoma patients treated with immunotherapy were accessed from Newell’s study (2).

### Patients’ collection at the First Affiliated Hospital of Zhengzhou University

For the in-house validation cohort, we retrospectively gathered formalin-fixed, paraffin-embedded (FFPE) tumor samples and clinical data from 104 melanoma patients who received PD-1/PD-L1 immune checkpoint blockade (ICB) therapy at the First Affiliated Hospital of Zhengzhou University (FAHZZU). Response assessment was prospectively conducted using RECIST 1.1 criteria. Patients were categorized as good responders if they had a RECIST complete response (CR), partial response (PR), or stable disease (SD) for more than 6 months; poor responders were those whose best response was RECIST progression or SD lasting 6 months or less. The detailed clinical information listed in **Supplementary Table S1**. The inclusion of patients and analysis of samples at FAHZZU were approved by the Institutional Review Board (IRB) of the First Affiliated Hospital of Zhengzhou University (Project ID 2024-KY-0801-002). All procedures in this study were conducted in accordance with the ethical standards of the institutional research committee and the 1964 Helsinki Declaration, along with its subsequent amendments.

### MYO1G DNA methylation and gene expression correlation analysis

Methylation data of 470 SKCM samples were generated by the Infinium HumanMethylation450 BeadChip (Illumina, San Diego, California, USA) and included about 450000 CpG site Beta values (considered as methylation level). The Beta value data for each sample was merged into a Beta-value matrix in which columns corresponded to samples and rows to CpGs. Then we used the Beta-mixture quantile normalization method wrapped in ChAMP R package (30) to normalize the Beta-value matrix. Genomic coordinate information of 9 CpG sites that are located on *MYO1G* gene body and promoter region were obtained and saved as a bed format file. We used Ensembl genome browser 109 to visualize CpG sites and genomic structure of *MYO1G* based on the bed file. The beta value of 9 CpG sites were extracted from normalized Beta-value matrix.

Normalized gene expression matrix generated by RNA-Seq analysis with fragments per kilobase of transcript per million mapped reads (FPKM), were downloaded from TCGA webpage. The gene expression value of *MYO1G* was extracted from expression data. To explore the potential epigenetic regulation of *MYO1G* gene expression via DNA methylation, we conducted Pearson correlation analysis between the 9 CpG sites and gene expression of *MYO1G*. CpG sites with absolute value of correlation coefficients >0.6 and p value < 0.01 were selected to stratify patients to high and low methylation groups with mean methylation value of these CpG sites.

### Whole transcriptome comparison analysis

The raw gene counts of 469 SKCM samples were downloaded from TCGA. Then we performed differential expression analysis between high and low methylation groups with DESeq2 (31). Genes with the Benjamini-Hochberg adjusted p value less than 0.05 and log2 fold-change more than 1.0 were considered differentially expressed.

### Gene set enrichment analysis

We collected 50 hallmark gene sets and 186 KEGG pathway signatures from Molecular Signatures Database (MSigDB v2022.1.Hs, https://www.gsea-msigdb.org/gsea/msigdb) (32). Based on high and low promoter methylation groups, gene set enrichment analysis was carried out to identify *MYO1G* promoter methylation-related gene sets with clusterProfiler (33). The predictive signatures of immunotherapy, such as a six-gene IFNγ signature (IFNγ-6), a related 18-gene expanded immune signature (IFNγ expanded immune 18) (34), an effector T cell signature (effector T-cell) (35) and a combined IFNγ Effector T-cell signature (36), were collected and used to calculate single sample gene set enrichment scores of each sample with the GSVA R package (37).

### Immune cell infiltration estimation

TIMER 2.0 web application (38) was used to estimate the correlations of *MYO1G* gene expression with the tumor purity and infiltration levels of B cell, CD8+ T cell, CD4+ T cell, macrophage, neutrophil and dendritic cell. The absolute fraction data of 22 infiltrating immune cells, which was inferred by the CIBERSORT algorithm (39) based on gene expression profiles, was downloaded from the TIMER database (38) (http://timer.cistrome.org/infiltration_estimation_for_tcga.csv.gz). And the leukocyte fraction data (TCGA_all_leuk_estimate.masked.20170107.tsv), which was estimated based on DNA methylation in Thorsson’s study (40), was retrieved from https://gdc.cancer.gov/about-data/publications/panimmune. The tumor-infiltrating lymphocyte (TIL) percentage evaluated by pathological images of TCGA tumors via deep learning method, is available in the supplementary table (**Supplementary Table S1**) in Saltz’s study (41). Then we compared immune cell infiltration, leukocyte fraction and TILs between high and low promoter groups.

For 104 melanoma patients who received PD-1/PD-L1 immune checkpoint blockade (ICB) therapy at the First Affiliated Hospital of Zhengzhou University (FAHZZU), digital histopathological whole slide images were scanned with the TEKSQRAY SQS-600P. We then used the cell segmentation and classification AI software Hover-Net (42) to perform lymphocyte count analysis based on the histopathological whole slide images.

### Quantitative methylation-specific PCR

**Figure 1:**
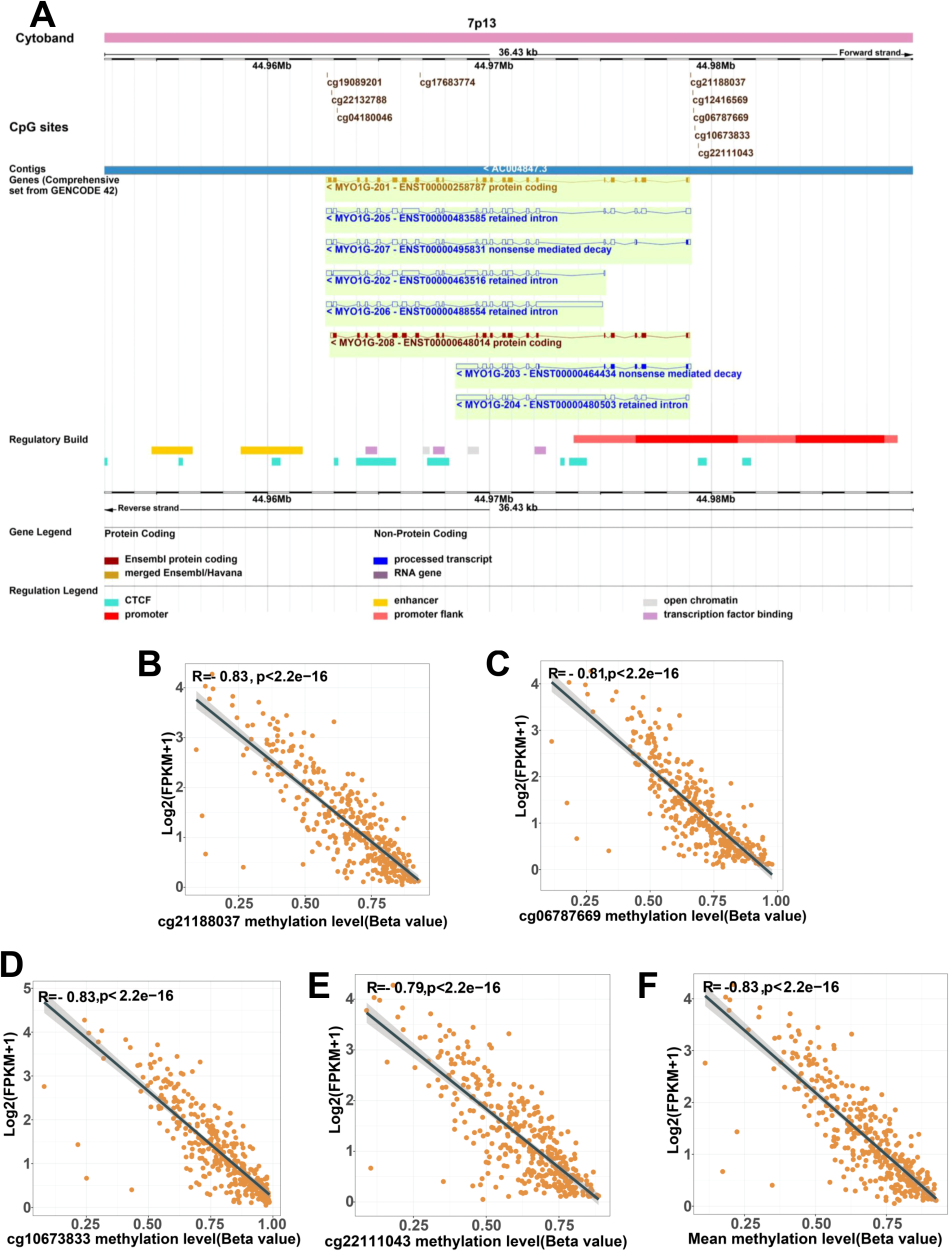
MYO1G promoter methylation is negatively associated with it’s gene expression in 366 metastatic melanomas from TCGA. **(A)** Overview of CpG sites and genomic structure of MYO1G. The illustration showing chromosome 7: 44,952,687-44,989,088 region, including MYO1G gene, its transcripts and regulatory elements (promoter, promoter flank, enhancer, CTCF, open chromatin and transcription fac tor binding); the illustration (modified) exported from www.ensembl.org (release 113). **(B-E)** Pearson correlations of MYO1G gene expression (log2 of normalized expression) with methylation levels of CpG sites. cg21188037 **(B)**, cg06787669 **(C)**, cg10673833 **(D)**, cg22111043 **(E)** among 358 metastatic melanoma patients. **(F)** Pearson correlation of MYO1G gene expression with mean methylation level of these four CpG sites. Pearson correlation analyses were performed using the stat_cor function from the ggpubr package in R.

Methylation analysis of the validation cohort of 105 melanoma patients from FAHZZU was conducted using bisulfite-specific quantitative real-time PCR, employing methylation-unspecific primers and probe pairs that specifically and competitively bind to methylated and unmethylated template DNA, respectively. This quantitative methylation-specific PCR (qMSP) is described in detail by Lehmann and Kreipe (43). Tumor tissue was macrodissected from FFPE blocks for DNA extraction with a Tissue DNA Kit (Amoy Diagnostics Co., Xiamen, China) and subsequently underwent bisulfite conversion using the EpiArt Magnetic DNA Methylation Bisulfite Kit (Nanjing Vazyme Biotech Co.,Ltd) according to the manufacturer’s protocol. We developed the qMSP assay targeting the CpG site as probed by Illumina HumanMethylation450 BeadChip bead cg22111043 and cg10673833 (**Figure 1**). Uncalibrated methylation levels, approximately considered percent methylation, were computed using cycle threshold (CT) values obtained from the probes specifically binding to methylated (CT methylated) and unmethylated (CT unmethylated) DNA, respectively (methylation [%]=100%/(1 + 2^CT methylated–CT unmethylated^). We performed 20 µL triplicate PCR reactions using a buffer composition containing 20 ng bisulfite converted DNA (quantified via UV-VIS spectrophotometry) and 0.2 µM each probe and 0.2 µM each primer (qMSP for cg22111043 CpG site forward primer: gttagagttatttgtgggattttaaaga, reverse primer: aattctaacttaaaaaacacacacaacc, probe methylated: 6-VIC- attttttgatgtgatt-MGB-1, probe unmethylated: FAM- ttttttgacgtgatt-MGB-1; qMSP for cg10673833 CpG site forward primer:gygttttataaggaggttgtgtttaa, reverse primer: ccaacraaaaaccctccaaaac, probe methylated: 6-VIC- aacatccaaccc-MGB-1, probe unmethylated: FAM-aacatccgacc-MGB-1; qMSP for cg06787669 CpG site forward primer: gttttagttttgggaggggttg, reverse primer: aatcaaaccrttaaacaacaacctc, probe methylated:6-VIC- ttataaaacacaaaaa-MGB-1, probe unmethylated: FAM-tataaaacgcaaaaa-MGB-1; qMSP for cg21188037 CpG site forward primer: gagagggggagggaaggttag, reverse primer: ccacttcctactttacaccaacact, probe methylated:6-VIC-caaaacaaaaac-MGB-1, probe unmethylated: FAM- caaaacgaaaac-MGB-1). qMSP was carried out using a Bio-Rad CFX96 real-time PCR detection system (Bio-Rad Laboratories, Hercules, CA) with the following temperature profile: 20 min at 95°C and 45 cycles with 50 s at 95°C, 50 s at 57°C and 50 s at 72°C.

### Targeted bisulfite sequencing assay

DNA extraction and bisulfite conversion were performed as previously described (59, 60). Based on the genomic coordinate of cg22111043 and cg10673833 CpG sites, we designed two paired primers (cg22111043 targeted bisulfite sequencing assay forward primer: tgggtttttttygttttgga, reverse primer: aaaaacacacacaaccaaataac; cg10673833 targeted bisulfite sequencing assay forward primer: ggggttgtttttygtatttgtta, reverse primer: aaaaaaccaacraaaaaccc) to detect it. The net-PCR was performed first to amplify the targeted DNA sequence. Then, the designed DNA fragments were sequenced by Illumina Hiseq 2000. BSseeker2, one of the most commonly used tools for analyzing the bisulfite sequencing results, was applied in our study for mapping bisulfite-treated reads as well as for methylation calling (61). After calling methylation, we obtained the bisulfite conversion rate for each sample, and samples with bisulfite conversion rate < 98% were firstly filtered out. After the preliminary analysis, we then calculated the average coverage as well as the missing rate for each CpG site. The CpG sites with average coverage less than 20× and/or with missing rate > 0.20 were further filtered out. In addition, the samples with missing rate > 0.30 were filtered out.

### Quantitative reverse transcriptase PCR

qRT-PCR was used to quantify *MYO1G* mRNA expression levels in 109 melanoma samples of the FAHZZU cohort. RNA extraction was performed using a FFPE DNA/RNA extraction Kit (AmoyDx, Xiamen, China) according to the manufacturer’s instructions. Complementary DNA (cDNA) was synthesized using 500 ng of total RNA using the HiScript III RT SuperMix for qPCR (+gDNA wiper) (Vazyme, Nanjing, China) according to the manufacturer’s instructions. The qRT-PCR assay was performed in 20 µl volumes using Taq Pro Universal SYBR qPCR Master Mix (Vazyme, Nanjing, China) containing 20 ng cDNA and 0.2 µM each primer (*MYO1G* forward primer: atcacctggcagagcgttgagt, *MYO1G* reverse primer: gattcggtcagtgatggtgcca). The housekeeping genes ACTB and GAPDH were used as references for normalization (ACTB forward primer: atgtggccgaggactttgatt, ACTB reverse primer: agtggggtggcttttaggatg; GAPDH forward primer: tgcaccaccaactgcttagc, GAPDH reverse primer: ggcatggactgtggtcatgag). qRT-PCR was carried out using a Bio-Rad CFX96 real-time PCR detection system (Bio-Rad Laboratories, Hercules, CA) with the following temperature profile: 30 s at 95°C and 40 cycles with 10 s at 95°C, and 30 s at 60°C. Relative *MYO1G* expression levels were calculated using the ΔCT method.

### Statistical analysis

All Statistical analyses were carried out in R environment. Correlations between two variables were calculated by Pearson correlation analysis with cor.test function in R. Continuous value comparisons between two groups were tested with Wilcoxon-Mann-Whitney U. Kaplan–Meier estimate and log-rank testing was used to conduct the survival analysis for overall survival (OS) and progression-free survival (PFS). The multi-variate Cox proportional hazard model was used to investigate the association of the combination of *MYO1G* promoter methylation, clinical tumor stage, age, and gender with survival.

## Results

### Association of *MYO1G* methylation and mRNA expression

DNA methylation is an important epigenetic mechanism involving the regulation of gene expression. The methylation of CpG island in promoter region results in the silencing of gene expression (44, 45). According to coordinate information of 9 CpG sites provided by the Infinium HumanMethylation450 BeadChip, we visualized the 9 CpG sites and genomic structure of *MYO1G* with Ensemble genome browser (**Figure 1A**). *MYO1G* had 8 transcripts, among which MYO1G-201 and MYO1G-208 were protein-coding transcripts and shared the same transcription start site. Near the transcription start site, the localization of a promoter and its flank was predicted. The 5 CpG sites were located in promoter region, and the remaining 4 CpG sites are located on gene body region. To investigate whether *MYO1G* expression is regulated by DNA methylation, we correlated the methylation levels of 9 CpG sites within the *MYO1G* gene with expression value among 358 melanoma samples from TCGA.

We observed significant inverse correlations between *MYO1G* gene expression and DNA methylation of the 5 CpG sites, such as cg21188037 (**Figure 1B**, R = -0.83), cg06787669 (**Figure 1C** *, R*=-0.81), cg10673833 (**Figure 1D**, R = -0.83), cg22111043 (**Figure 1E**, R = -0.79), cg12416569 (**Supplementary Figure 1E**, *R* = -0.46). The methylation level of 4 CpG sites located on gene body were positively correlated with *MYO1G* gene expression, but the Pearson correlation coefficients of them were less than 0.4 (**Supplementary Figures 1A–D**). To understand the impact of promoter methylation on gene expression, we selected 4 CpG sites (cg21188037, cg06787669, cg10673833 and cg22111043) with R < -0.6 to calculate the mean methylation level of these CpG sites, and defined it as the promoter methylation level of *MYO1G*. In conclusion, the hypermethylation of CpG sites in *MYO1G* promoter may lead to the silencing of gene expression.

### Prognostic value of *MYO1G* promoter methylation and gene expression

**Figure 2:**
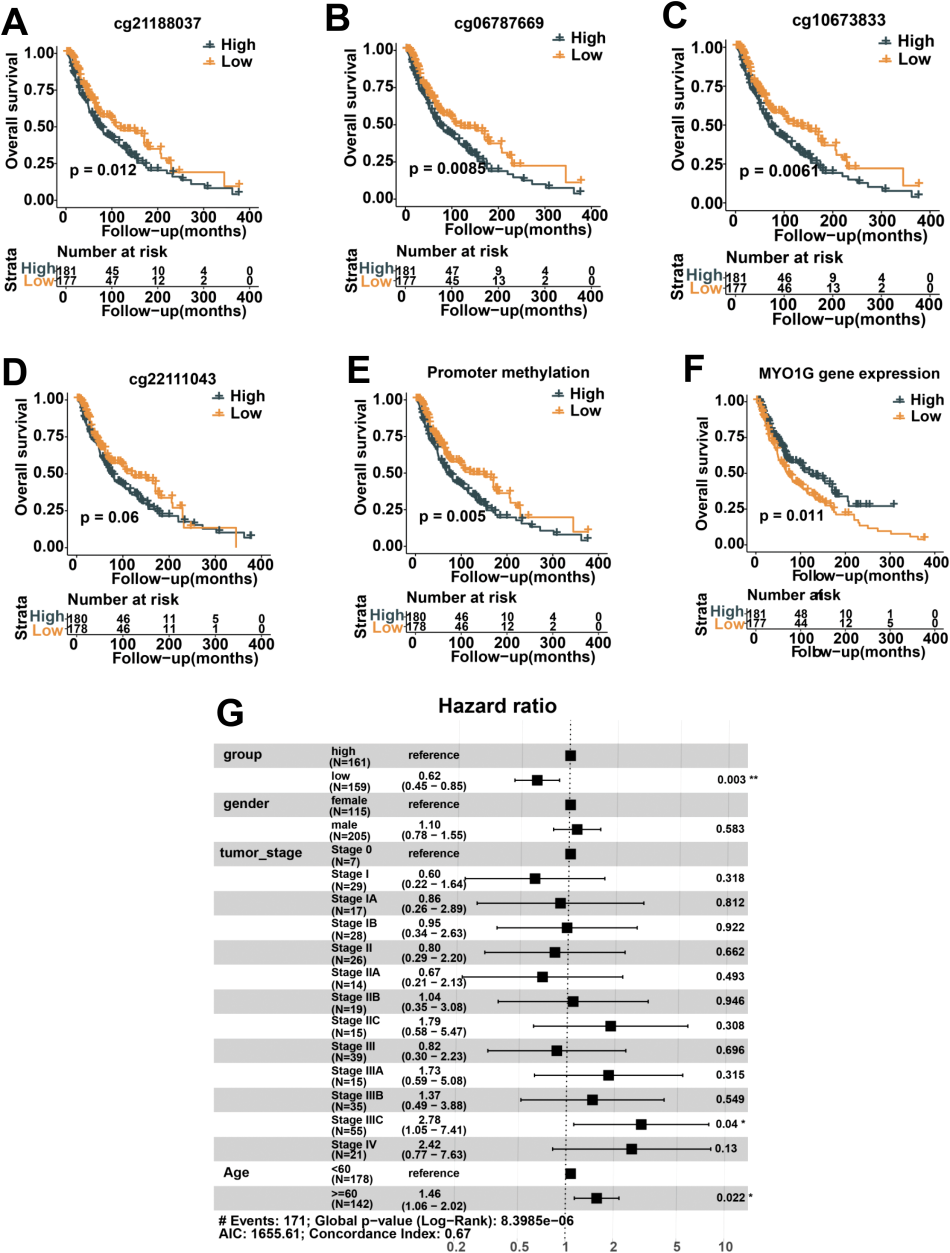
Prognostic analysis in 358 metastatic melanoma patients from TCGA stratified according to MYO1G methylation and gene expression. **(A-D)** Kaplan-Meier curves of overall survival in melanoma patients stratified according to median Beta values of four CpG sites. **(E)** Kaplan-Meier curves of overall survival in these patients stratified according to median promoter methylation defined by mean Beta value of four CpG sites. **(F)** Kaplan-Meier curves of overall survival in these patients stratified according to MYO1G gene expression. **(G)** Multivariate Cox regression analysis on four variables (group[high/low promoter methylation group], gender, tumor stage, and age). All Kaplan-Meier survival analyses were performed using the R package survminer. *P < 0.05; **P < 0.01.

To investigate whether *MYO1G* promoter methylation and gene expression could predict the prognosis of melanoma, we stratified the 358 metastatic SKCM into two group (high and low groups) based on Beta values of four CpG sites and FPKM of *MYO1G* respectively, and correlated the two groups with overall survival. We observed that hypomethylation of cg21188037 (**Figure 2A** *, P=0.012*), cg06787669 (**Figure 2B** *, P*=0.0085), cg10673833 (**Figure 2C**, P = 0.0061) and cg22111043 (**Figure 2D**, P = 0.06) were significantly associated with prolonged overall survival time. Based on *MYO1G* promoter methylation defined by the mean methylation level of the 4 CpG sites, we also observed the melanoma patients belonging to low methylation group have longer overall survival (*P*=0.005) than those belonging to high methylation group (**Figure 2E**). The promoter hypomethylation of *MYO1G* mirrored high gene expression level of this gene. As expected, we also found that increased expression of *MYO1G* was associated with prolonged overall survival time (**Figure 2F**). We integrated multiple variables including the tumor stage, gender, age and promoter methylation into the Cox proportional-hazards model to investigate the independent prognostic value of promoter methylation, and found that the hypomethylation of promoter remained an independent prognostic factor with hazard ratio=0.62 and P value=0.003 (**Figure 2G**). Overall, *MYO1G* promoter hypomethylation correlates with increased mRNA expression, both of which can be used as predictive biomarkers of favorable prognosis.

### Associations of *MYO1G* promoter methylation and gene expression with immune cell infiltration in TME

**Figure 3:**
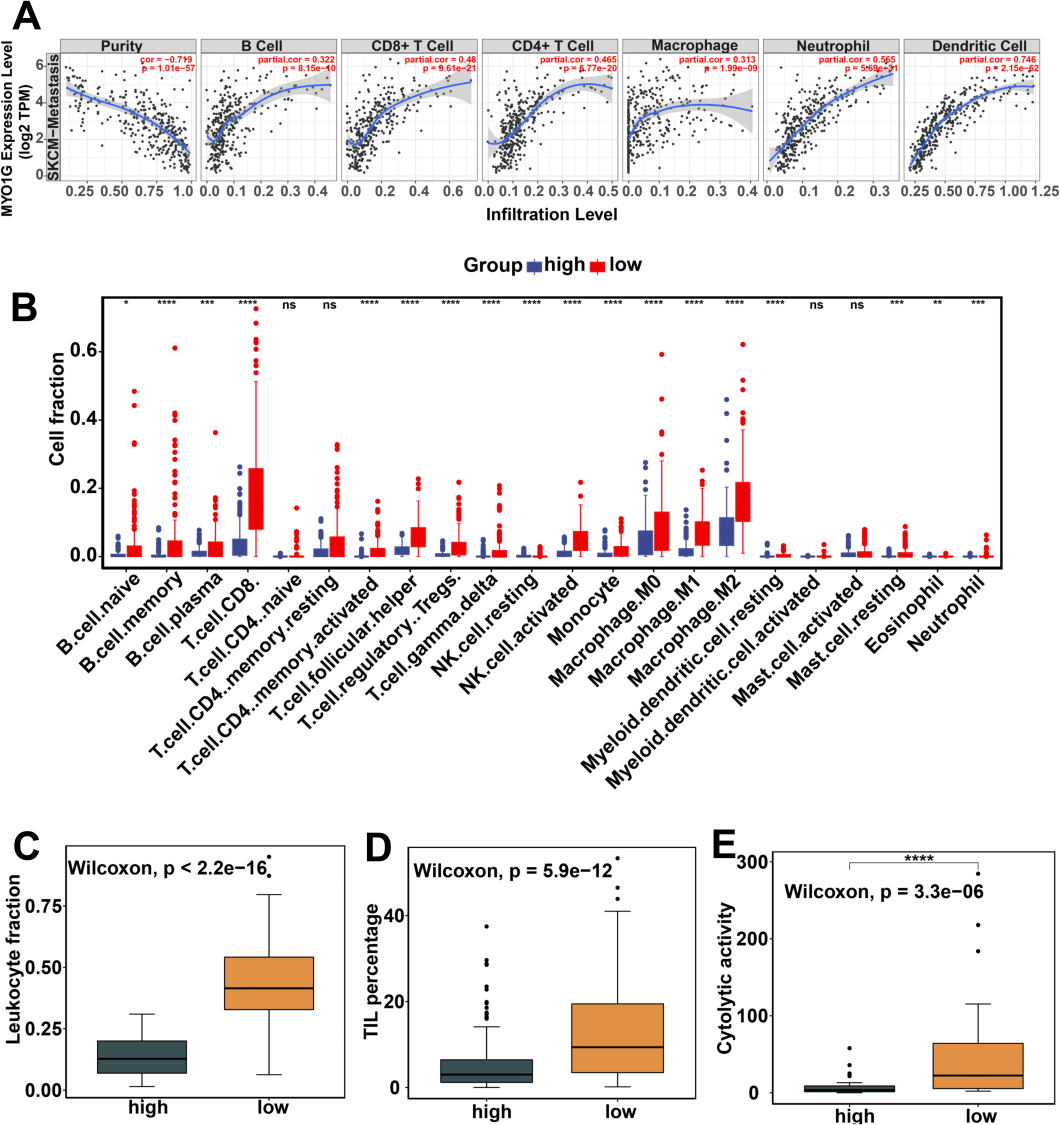
Correlations of MYO1G gene expression and promoter methylation with immune cell infiltration among 366 metastatic melanomas from TCGA. **(A)** the correlations of MYO1G gene expression with tumor purity and the infiltration levels of immune cells (B cell, CD8+ T cell, CD4+ T cell, Macrophages, Neutrophil and Dendritic cells) estimated by TIMER2.0(http://timer.cistrome.org/). **(B)** The comparison of the absolute fraction of TME cells between the high and low promoter methylation groups. **(C-E)** Box plots show the differences of leukocyte fraction **(C)**, TIL percentage **(D)** and cytolytic activity **(E)** between two groups. All statistical differences of two classes were compared by Wilcoxon rank-sum test; *P < 0.05; **P < 0.01; ***P < 0.001; ****P < 0.0001. ns, no significance.

Tumor-infiltrating Immune cells have been reported to be independent predictors of prognosis (46). Therefore, we investigated whether *MYO1G* promoter methylation and gene expression impacted the immune cell infiltration in TME of metastatic SKCM. TIMMER 2.0 web application analysis results indicated that increased *MYO1G* gene expression was inversely correlated with tumor purity (**Figure 3A**), suggesting that *MYO1G* may play an important role in controlling tumor growth. When looking at immune cells, *MYO1G* gene expression showed a positive correlation with the infiltration level of B cell, CD8+ T cell, CD4+ T cell, macrophage, neutrophil and dendritic cell (**Figure 3A**), indicating that *MYO1G* may impact lymphocytes infiltration. To explore whether MYO1G promoter methylation was also linked with immune cell infiltration, we compared the 22 immune cells infiltration level estimated by CIBERSORT (39) between high and low *MYO1G* promoter methylation groups of metastatic SKCM patients. We found that 15 immune cell types were significantly enriched in the low promoter methylation group (**Figure 3B**), among which CD8 T cell (21), memory B cell, naïve B cell (47) and M1 macrophage were previously reported to be associated with favorable prognosis of SKCM. leukocyte fraction (**Figure 3C**) imputed by methylation data and tumor-infiltrating lymphocyte percentage (**Figure 3D**) evaluated by pathological images were also higher in the low promoter methylation group. The cytolytic activity defined as the geometric mean of *GZMA* and *PRF1* expression value is associated with anti-tumor immune response and prognosis (48). We found that the low promoter methylation group had a higher the cytolytic activity score than high methylation group (**Figure 3E**). These results indicated that hypomethylation of *MYO1G* promoter and high *MYO1G* mRNA expression was associated with high immune cell infiltration level, and subsequently impacted prognosis.

### Alterations in immunological molecular characteristics related to *MYO1G* promoter methylation

**Figure 4:**
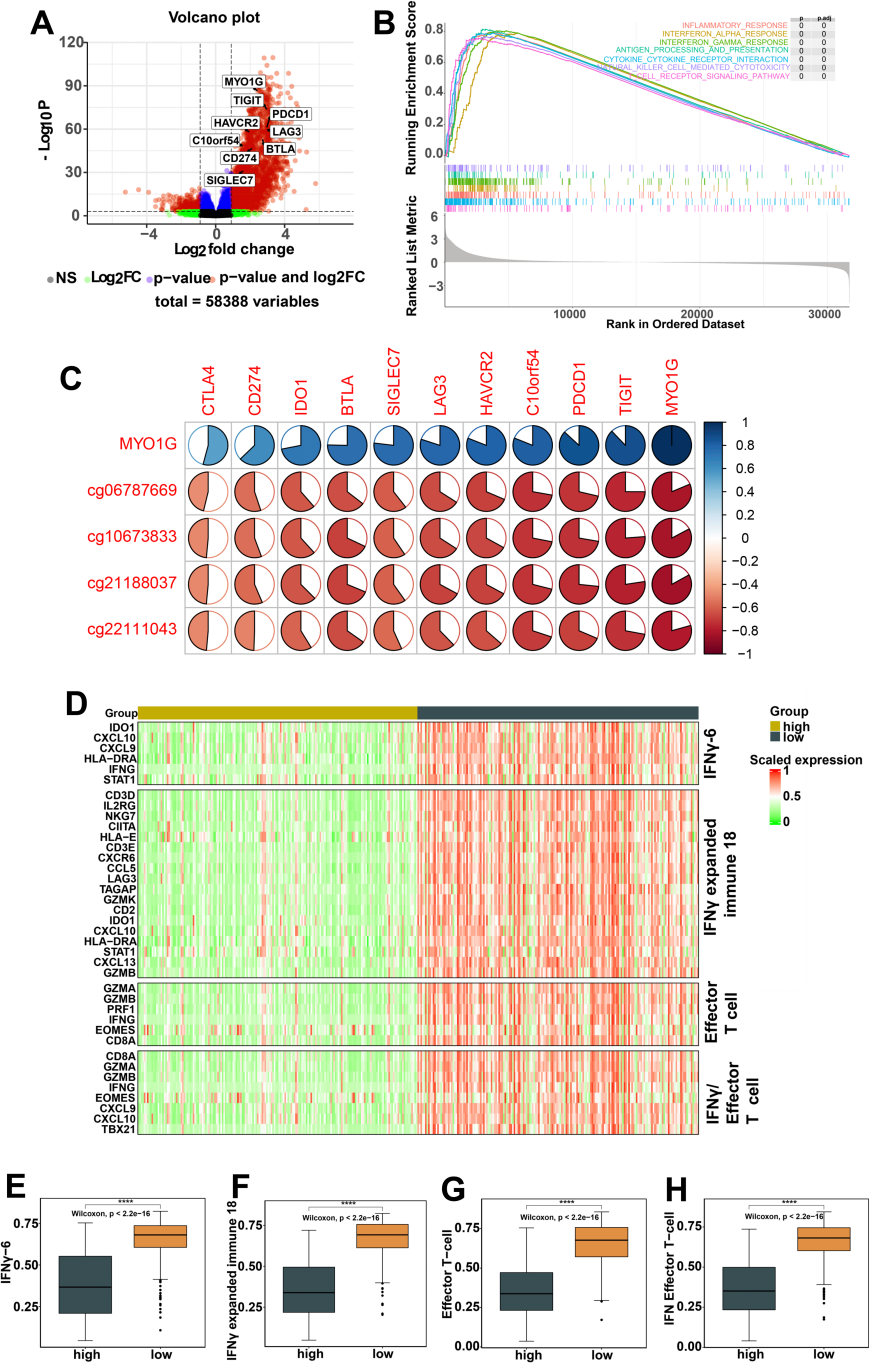
Molecular and pathway alterations related to MYO1G promoter methylation. **(A)** Volcano plot showing differentially expressed genes between high and low promoter methylation groups of 358 metastatic melanomas from TCGA. Genes with log2 fold change > 1 were considered to be up-regulated in low promoter methylation group. **(B)** Unbiased gene set enrichment analysis with clusterProfiler of 3 hallmark gene sets and 4 KEGG pathways, considered enriched (adjusted p value < 0.01)in low promoter methylation group. **(C)** The correlations of MYO1G gene expression and the Beta values of four CpG sites with immune checkpoint genes. The figure generated by R package corrplot. **(D)** The heatmap showing gene expression level for gene sets with a significant difference (Mann-Whitney U test, p < 0.05) between high and low promoter methylation groups. **(E-H)** Box plots of gene set enrichment scores. Box plots show the median, first and third quartiles and the whiskers extend to 1.5 times the interquartile range and p values were calculated using two-sided Wilcoxon rank sum. **(E)** IFNγ-6. **(F)** IFNγ expanded immune 18. **(G)** Effector T cell. **(H)** IFNγ/Effector T cell. All statistical differences of two classes were compared by Wilcoxon rank-sum test with R software; ****P < 0.0001.

To reveal transcriptomic molecular characterizations associated with *MYO1G* promoter methylation, differential expression analysis was performed between high and low promoter methylation groups of 366 metastatic SKCM. 3049 genes were identified to be significantly upregulated in the low promoter methylation group (**Supplementary Table S2**), including 8 immune checkpoint genes (*TIGIT*, *PDCD1*, *LGA3*, *BTLA*, *HAVCR2*, *CD274*, *C10orf54* and *SIGLEC7[P <0.01]*) (**Figure 4A**). Unbiased gene set enrichment analysis demonstrated that 9 hallmark gene sets and 39 KEGG pathways (FDR <0.05) were significantly enriched in the low promoter methylation group (**Supplementary Table S3**). Of note, the anti-tumor immune response-related hallmark gene sets and KEGG pathways including inflammatory response, interferon alpha response, interferon gamma, antigen processing and presentation, cytokine-cytokine receptor interaction, natural killer cell medicated cytotoxicity, T cell receptor signaling pathway, were significantly enriched in the low methylation group (**Figure 4B**, FDR < 0.001). Additionally, we observed a significant inverse correlation between the methylation level of four CpG sites and the expression of immune checkpoint genes. *MYO1G* gene expression was significantly and positively correlated with the expression of immune checkpoint genes (**Figure 4C**, P < 0.001), suggesting that *MYO1G* promoter methylation regulated gene expression and subsequently impacted anti-tumor immune response. Furthermore, we explored the expression of four gene sets previously reported to be associated with immunotherapy response, and found that these genes were significantly up-regulated in the low methylation group (**Figure 4D**). Gene set enrichment scores of these gene sets, including a six-gene IFNγ signature (34) (**Figure 4E**), a related 18-gene IFNγ signature (34) (**Figure 4F**), an effector T cell signature (35) (**Figure 4G**), a combined IFNγ/effector T cell signature (36) (**Figure 4H**) were significantly higher in low methylation group compared with high methylation group.

### Correlation of *MYO1G* promoter methylation with gene expression and immune cell infiltration in primary SKCM

**Figure 5:**
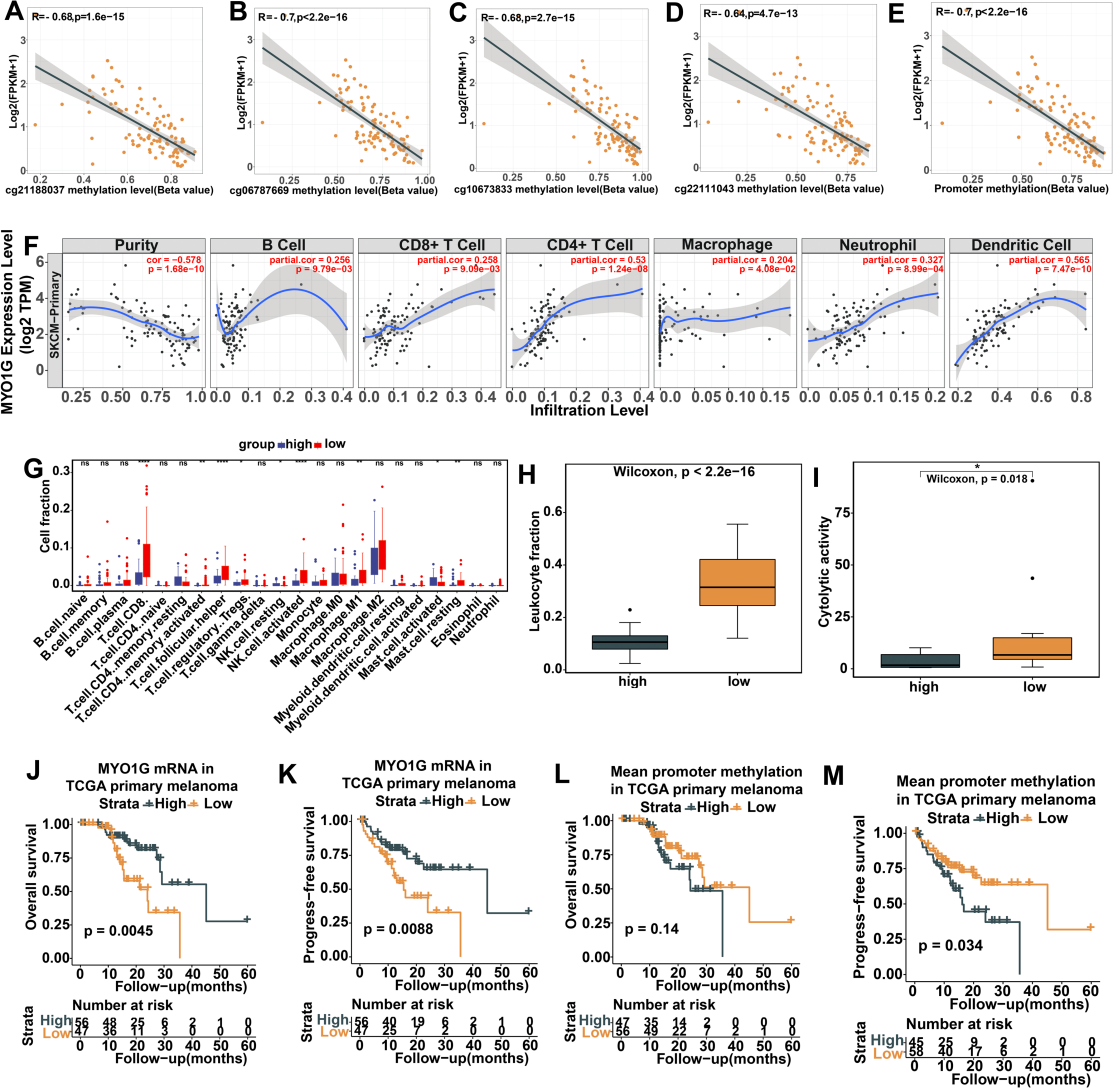
Validation of the correlations between MYO1G promoter methylation, gene expression, immune cell infiltration and prognosis in 103 primary melanomas from TCGA. **(A-D)** Pearson correlations between the Beta value of four CpG sites and MYO1G gene expression. **(E)** Pearson correlations between MYO1G promoter methylation and it's expression. **(F)** The correlations of MYO1G gene expression with the infiltration levels of immune cells (B cell, CD8+ T cell, CD4+ T cell, Macrophages, Neutrophil and Dendritic cells). **(G)** The comparison of the absolute fraction of TME cells between the high and low promoter methylation groups. **(H, I)** Box plots show the differences of leukocyte fraction **(H)** and cytolytic activity **(I)** between two groups. All statistical differences of two classes were compared by Wilcoxon rank-sum test; *P < 0.05; **P < 0.01; ****P < 0.0001. **(J, K)** Kaplan-Meier analysis of overall survival **(J)** and progression-free survival **(K)** based on MYO1G gene expression. **(L, M)** Kaplan-Meier analysis of overall survival **(L)** and progression-free survival **(M)** based on MYO1G promoter methylation. All Kaplan-Meier survival analyses were performed using the R package survminer. ns, no significance.

To validate the correlations of *MYO1G* promoter methylation with biological, immune features and prognosis in SKCM, we conducted the same analysis on 103 primary SKCM samples from the TCGA. Consistent with metastatic SKCM, the methylation levels of cg21188037 (*R*=-0.68, **Figure 5A**), cg06787669 (*R*=-0.7, **Figure 5B**), cg10673833 (*R*=-0.68, **Figure 5C**), and cg22111043 (*R*=-0.64, **Figure 5D**) were significantly and inversely correlated with *MYO1G* gene expression. As expected, the mean methylation level of these CpG sites showed a significant inverse correlation with *MYO1G* gene expression (*R*=-0.7, **Figure 5E**). When we examined immune cell infiltration, we also observed significant positive correlations between *MYO1G* gene expression and the infiltration levels of B cells, CD8+ T cells, CD4+ T cells, macrophages, neutrophils, and dendritic cells (**Figure 5F**). Based on the mean methylation level of the four CpG sites, we divided the 103 primary SKCM samples into high and low methylation groups. Similar to metastatic SKCM, we found that the cell fractions of multiple immune cell types, such as CD8+ T cells, activated memory CD4+ T cells, T follicular helper cells, activated NK cells, and M1 macrophages, were significantly higher in the low methylation group than in the high methylation group (**Figure 5G**). The leukocyte fractions estimated by DNA methylation (**Figure 5H**) and cytolytic activity scores (**Figure 5I**) were higher in the low methylation group. We also investigate prognostic significance of MYO1G expression and promoter methylation in 103 TCGA primary SKCM patients. Consistent with metastatic SKCM, we observed that higher *MYO1G* gene expression was notably associated with longer overall survival (**Figure 5J**, *P*=0.0045) and progression-free survival (**Figure 5K**, *P*=0.0088) in primary SKCM. With regard to *MYO1G* promoter methylation, although the overall survival didn’t show a significant difference between high and low methylation groups (**Figure 5L**, *P*=0.14), the progression-free survival of the low methylation group was significantly longer than that of the high methylation group (**Figure 5M**, *P*=0.034).Overall, our findings based on 103 primary SKCM samples further validate significant correlations of *MYO1G* promoter hypomethylation with elevated gene expression, enhanced immune cell infiltration and favorable prognosis in SKCM.

### The correlation of MYO1G expression and promoter methylation with prognosis and immunotherapy response in independent melanoma cohorts

**Figure 6:**
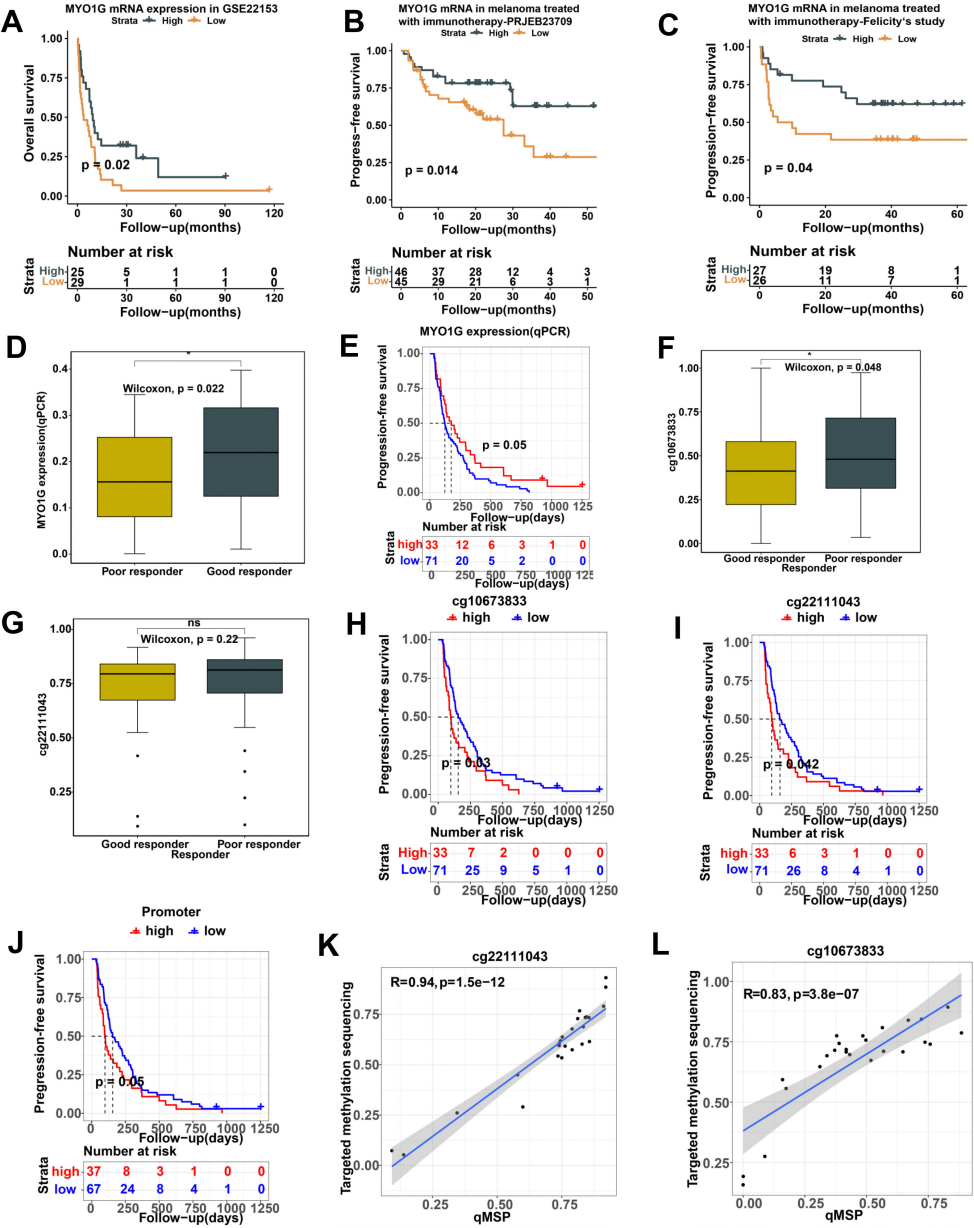
Validation of prognostic values of MYO1G gene expression and promoter methylation in independent melanoma cohorts. **(A)** Kaplan-Meier analysis of overall survival based on MYO1G gene expression in 54 melanoma patients from GEO databased with accession number GSE22153. **(B)** Kaplan-Meier analysis of overall survival based on MYO1G gene expression in 94 melanoma patients treated with immunotherapy from European Nucleotide Archive database with accession number PRJEB23709. **(C)** Kaplan-Meier analysis of progression-free survival based on MYO1G gene expression in 53 melanoma patients treated with immunotherapy from Felicity’s study. **(D)** Box plot show differential expression level of MYO1G quantified by qPCR in our FAHZZU melanoma cohort. **(E)** Kaplan-Meier analysis of progression-free survival based on MYO1G expression (qPCR) in our FAHZZU melanoma cohort. **(F, G)** Box plots show differential methylation level of cg10673833 **(F)** and cg22111043 **(G)** between good and poor responders in our FAHZZU melanoma cohort. **(H-J)** Kaplan-Meier analysis of progression-free survival based on the methylation levels of cg22111043 **(H)**, cg10673833 **(I)** CpG sites and promoter **(J)** in 104 melanoma patients treated with immunotherapy from our FAHZZU melanoma cohort. **(K, L)** The correlations between the methylation levels of cg22111043 **(K)** and cg10673833 **(L)** CpG sites quantified by qMSP and those quantified by targeting methylation sequencing. All Kaplan-Meier survival analyses were performed using the R package survminer. All statistical differences of two classes were compared by Wilcoxon rank-sum test; *P < 0.05.

Furthermore, we conducted an overall survival analysis on 54 stage IV SKCM patients based on *MYO1G* gene expression and confirmed that high *MYO1G* gene expression was correlated with prolonged overall survival (**Figure 6A**, *P*=0.02). To investigate whether *MYO1G* expression can be a predictor of immunotherapy response, we carried out an overall survival analysis on 91 SKCM patients treated with immunotherapy. The result indicated that patients with high *MYO1G* gene expression had a better prognosis than those with low *MYO1G* gene expression (**Figure 6B**, *P*=0.014). We further verified the prognostic value of *MYO1G* gene expression in a melanoma cohort treated with immunotherapy from Newell’s study (2). We also found that high *MYO1G* mRNA expression level was significantly associated with prolonged progression-free survival among 53 melanoma patients treated with immunotherapy (**Figure 6C**, *P*=0.04). Our comparative analysis in the FAHZZU melanoma cohort showed that patients who responded well to immunotherapy had higher MYO1G expression levels (**Figure 6D**), and those with elevated expression also exhibited longer progression-free survival (**Figure 6E**). We also performed methylation and expression analysis on 104 melanoma patients from our FAHZZU cohort. We found a significant negative correlation between the methylation levels of cg22111043(R=-0.42, *P <*0.001) and cg10673833(R=-0.26, *P <*0.001) CpG sites quantified by the qMSP assay and the *MYO1G* gene expression quantified by the qPCR. But, there are no significant correlations between the methylation levels of cg06787669 (R=-0.049, *P* =0.38) and cg21188037 (R=-0.039, *P* =0.69) CpG sites quantified by the qMSP assay and the *MYO1G* gene expression quantified by the qPCR. Therefore, we focused on cg22111043 and cg10673833 CpG sites. We compared the methylation level of cg10673833 and cg22111043 between good responders and poor responders in our FAHZZU cohort treated with immunotherapy. Then we found that good responders have significantly lower methylation level of cg10673833 than poor responders (**Figure 6F**, P=0.048).With regard to cg22111043, the good responders tend to show lower methylation level of this CpG site than poor responders (**Figure 6G**). We further analyzed the correlations between the methylation levels of the cg22111043 (**Figure 6H**, P=0.03) and cg10673833 (**Figure 6I**, P=0.042) sites, and progression-free survival (PFS) and found that immunotherapy patients with lower methylation levels exhibited significantly longer PFS. When we looked at the promoter methylation level defined by mean value of cg22111043 and cg10673833, we also found the patients with lower methylation levels showed prolonged PFS (**Figure 6J**, P=0.06). To validate the accuracy of qMSP quantification for these sites, we performed targeted methylation sequencing and correlated the methylation levels of cg22111043 (**Figure 6K**, R=0.94, P<0.0001) and cg10673833 (**Figure 6L**, R=0.83, P<0.0001) CpG sites calculated by targeted bisulfite sequencing assay with that by qMSP. The results demonstrated a strong consistency between the methylation levels measured by qMSP and those obtained through targeted methylation sequencing.

### Validation of correlation between MYO1G promoter hypomethylation and lymphocyte infiltration in FAHZZU melanoma cohort

**Figure 7:**
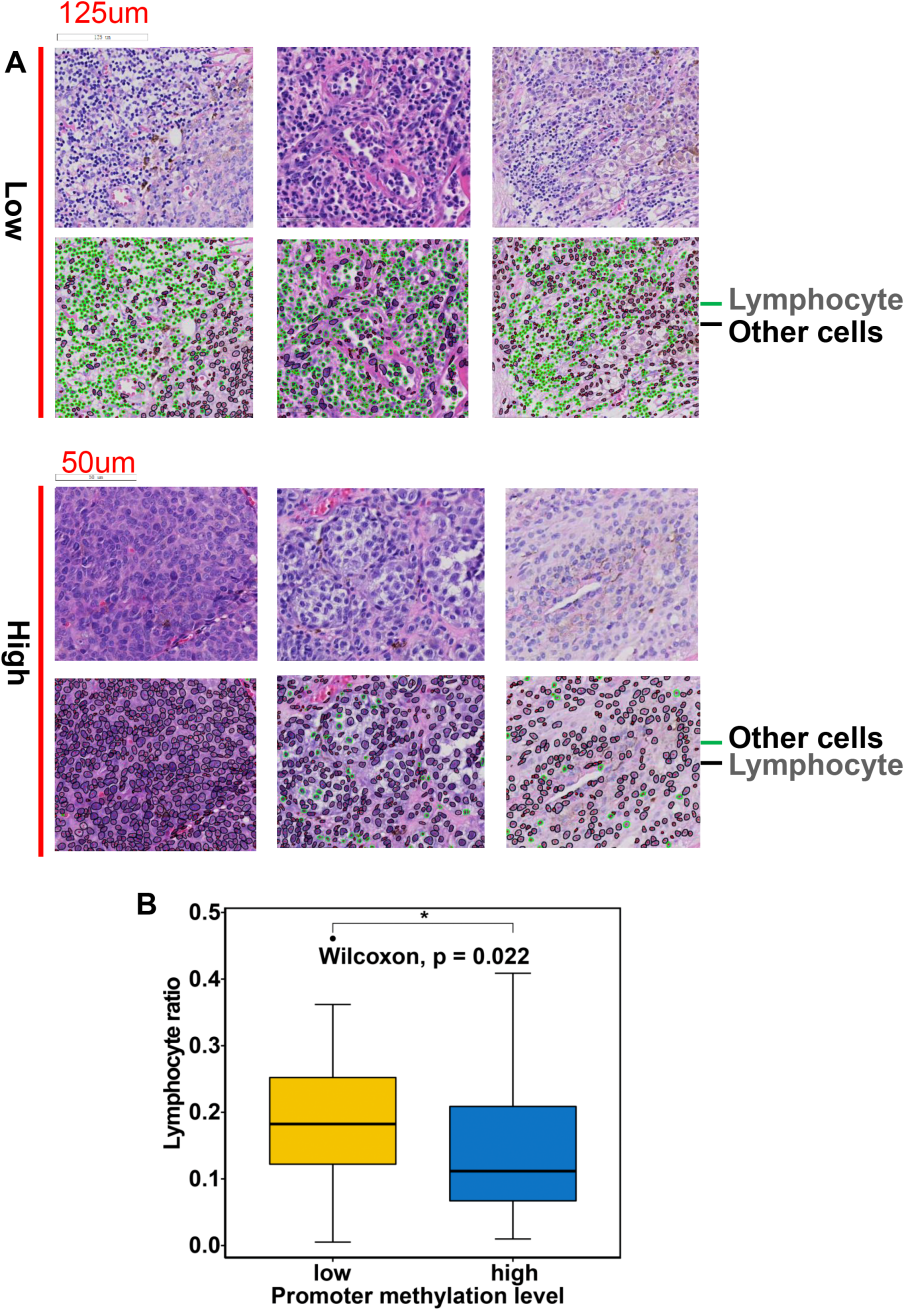
The lymphocyte infiltration analysis based on whole-slide digital pathology images from 104 melanoma patients in the FAHZZU cohort. **(A)** The HE stained pathology images show the infiltration level of lymphocytes in high and low methylation groups. Lymphocytes are labeled in green, and other cells are labeled in black with Hover-Net. **(B)** Box plot shows difference of lymphocyte ratio between high and low methylation groups with R package ggpubr. All statistical differences of two classes were compared by Wilcoxon rank-sum test; *P < 0.05.

To assess the level of lymphocyte infiltration, we used the AI-based cell segmentation and classification software Hover-Net to perform cell count and statistical analysis on the whole-slide images of pathology from 104 melanoma patients. Based on the cell classification results, we visualized the types of cells present in the pathology images, with lymphocytes marked in green and other cell types marked in black (**Figure 7A**). We then calculated the proportion of lymphocytes in each pathology image and compared the lymphocyte proportions between the low and high MYO1G promoter methylation groups. The results showed that patients in the low MYO1G promoter methylation group had significantly more lymphocyte infiltration (**Figure 7B**). Theses results demonstrated that MYO1G promoter hypomethylation was associated with elevated lymphocyte infiltration.

## Discussion

In this study, we explored the epigenetic regulation of *MYO1G* gene expression through the DNA methylation in the promoter region and evaluated the *MYO1G* promoter methylation and gene expression as biomarkers associated with immunotherapy response, overall survival, progression free survival and immune cell infiltration in melanoma. Our results suggested that *MYO1G* gene expression was regulated by promoter methylation. Our findings demonstrated that promoter hypomethylation was significantly associated with increased gene expression of *MYO1G*, prolonged overall and progression free survival, and enhanced immune cell infiltration in melanoma. Overall, our study confirmed that *MYO1G* promoter methylation and gene expression were predictive biomarkers for immune cell infiltration in the tumor microenvironment, prognosis and immunotherapy response of cutaneous melanoma patients.

The abnormal alterations in DNA methylation were involved in many human diseases (49). Aberrant promoter methylation was considered an important epigenetic mechanism involved in silencing of gene expression in human cancers (50, 51). Our study firstly identified significant inverse correlations of *MYO1G* gene expression with methylation level of 4 CpG sites including cg21188037, cg06787669, cg10673833 and cg22111043 in 358 metastatic melanoma samples, which were located on *MYO1G* promoter region. As expected, the mean methylation level of the four CpG sites was negatively associated with *MYO1G* gene expression. Then we robustly validated the correlations of promoter methylation with *MYO1G* gene in two cohorts including the 103 primary melanoma samples from TCGA and 104 advanced melanoma samples from this study. These findings strongly support the notion of epigenetic regulation of *MYO1G* gene expression via it’s promoter DNA methylation.

The detection of DNA methylation has gradually become a novel paradigm for tumor diagnosis and prognostic prediction (52). Genome-wide methylation interrogation with machine learning methods has allowed for clinical-grade classifier development in the central nervous system and soft tissue tumors. Methylation of the *MGMT* promoter is utilized as a biomarker to predict clinical response to a chemotherapeutic agent in glioma (53).With regard to *MYO1G*, cg10673833 located on *MYO1G* promoter region was reported to be a biomarker in the diagnosis and disease monitoring of colorectal cancer (22). Additionally, hypermethylation of *MYO1G* gene is a potential diagnostic biomarker for hepatocellular carcinoma (23). However, the clinical significance of *MYO1G* promoter methylation in melanoma is still unknown. In this study, we first demonstrated that the hypomethylation of cg21188037, cg06787669, cg10673833 and cg22111043 were significantly associated with favorable prognosis in the metastatic melanoma cohort of 358 patients. As expected, *MYO1G* promoter hypomethylation defined as the mean methylation level of four CpG sites was significantly correlated with prognosis. Furthermore, the multiple variable COX regression model integrating gender, age, tumor stage and promoter methylation, indicates that promoter hypomethylation remains independently predictive of favorable prognosis in metastatic melanoma. We also validated that the *MYO1G* promoter hypomethylation could predict prolonged progress-free survival in primary melanoma. Owing to the limited sample size, we were unable to observe a significant association between *MYO1G* promoter methylation and progression-free survival in melanoma patients receiving immunotherapy. However, patients with promoter hypomethylation tended to exhibit prolonged progression-free survival. Our study validated that *MYO1G* gene expression was negatively correlated with promoter methylation. In contrast to promoter methylation, higher *MYO1G* gene expression can predict a better prognosis in melanoma. The prognostic value of *MYO1G* gene expression was robustly validated in 103 primary melanomas from TCGA and 54 stage IV melanomas from GSE22153. Additionally, *MYO1G* gene expression was validated to be associated with a favorable prognosis of 91 patients treated with immunotherapy.

Immune cell infiltration in melanoma tumor microenvironment is significantly associated with prognosis and immunotherapy response. Specifically, ‘immune-cold’ tumors without T lymphocytes infiltration don’t respond to immune checkpoint blockade-based immunotherapy (3). Previous studies demonstrated that MYO1G plays an important role in the regulation of lymphocyte migration (14, 17). However, the biological significances of *MYO1G* gene expression and promoter methylation in tumors are still unclear. In this study, we demonstrated that *MYO1G* gene expression is positively correlated with infiltration levels of lymphocyte cells including B cells, CD8+ T cells, neutrophils and dendritic cells in primary and metastatic melanoma The accumulated evidence suggests that B cells (54), M1 macrophage (2) and CD8+ T cell infiltration (55) in melanoma are critical factors for predicting prognosis and immunotherapy response (7, 20), therefore, the positive correlations of *MYO1G* expression with infiltration levels of these immune cells support the prognostic value of *MYO1G*. Intriguingly, the correlation coefficient between dendritic cell infiltration levels and *MYO1G* expression exceeds 0.7, which reinforces the findings of prior research suggesting that *MYO1G* plays a crucial role in enhancing the interactions between T cells and dendritic cells during lymph node surveillance (14). On the other hand, tumor purity defined as the percentage of cancer cells (56, 57) is negatively correlated with *MYO1G* expression, suggesting that a high expression level of *MYO1G* may promote anti-tumor immune response to suppress tumor cell proliferation in melanoma. With regard to methylation, the melanoma patients were divided into high and low methylation group based on *MYO1G* promoter methylation level. The low methylation group with high *MYO1G* expression had higher cell fractions of immune cells including CD8+ T cell, M1 macrophage, activated NK cell and so on. Leukocyte fraction estimated by the genome-wide methylation profile and the evaluated tumor-infiltrating lymphocyte (TIL) percentage through pathological images are also higher in the low methylation group. Cytolytic activity which is associated with anti-tumor immune responses and improved prognosis (48), is significantly higher in the low methylation. A high density of lymphocyte infiltration (4, 5) is associated with favorable prognoses such as longer progression-free survival or improved overall survival. In line with previous studies, our study demonstrates that melanoma patients belonging to low promoter methylation group with high infiltration of lymphocytes and enhanced cytolytic activity showed favorable prognosis. In summary, *MYO1G* promoter hypomethylation may regulate gene expression, then promote immune cell migration to tumors and intrigue anti-tumor immune response, resulting in improved prognosis of patients.

To reveal specific immune-related molecule and pathway alterations which are impacted by *MYO1G* promoter methylation and gene expression, whole-transcriptome comparison analysis between high and low promoter methylation groups is performed and we identified significant upregulation of multiple immune checkpoint genes and *MYO1G* in low methylation group. Of note, *TIGIT (* 58), *PDCD1 (* 59), *HAVCR2 (* 60), *CTLA4 (* 61) and *BTLA (* 62) expression were reported to be predictive for favorable prognosis and immunotherapy response in melanoma. Furthermore, we demonstrate the positive correlations of *MYO1G* expression and the negative correlations of promoter methylation with expression of immune checkpoint genes, supporting that *MYO1G* expression and promoter methylation may be associated with favorable prognosis and immunotherapy response. Furthermore, four gene signatures for predicting immunotherapy response, including a six-gene IFNγ signature (34), a related 18-gene IFNγ signature (34), an effector T cell signature (35), a combined IFNγ/effector T cell signature (36), were significantly up-regulated in melanoma patients belonging to low promoter methylation group with high *MYO1G* gene expression level, supporting that *MYO1G* expression can predict prognosis in melanoma patients treated with immune checkpoint blockade.

Our findings suggest that *MYO1G* promoter hypomethylation is associated with increased *MYO1G* expression and elevated immune infiltration in melanoma. Interestingly, we also observed correlations between *MYO1G* methylation status and the expression of key immune checkpoint genes such as *TIGIT*, *PDCD1* and *LAG3*, which are canonical markers of CD8^+^ T cell exhaustion. This raises the possibility that *MYO1G* hypomethylation may not only reflect a more active immune microenvironment but also coexist with an exhausted T cell phenotype. One possible explanation is that elevated *MYO1G* expression in immune cells may participate in shaping the tumor immune landscape, potentially promoting T cell infiltration while simultaneously being involved in chronic stimulation and exhaustion signaling pathways. This duality has been observed in other cancers, where hypomethylation of immune-related genes correlates with both immune activation and immune regulatory mechanisms (63–65). Given that *MYO1G* is expressed in T cell or B cell (14, 17, 18), and involved in actin remodeling and membrane dynamics, its expression may influence T cell migration or interaction with antigen-presenting cells, thereby affecting exhaustion status. Notably, actin cytoskeletal regulators have been shown to modulate immune synapse stability and T cell receptor signaling, which are critical for sustaining T cell function versus exhaustion (13). Further experimental validation, such as single-cell methylation or co-localization of *MYO1G* with exhausted T cell subsets in melanoma tissue, would help clarify this relationship.

Our study has some limitations. While we demonstrated that *MYO1G* promoter hypomethylation may regulate gene expression and immune cell infiltration in melanoma patients based on multi-omics data from TCGA, it is important to acknowledge that these observations are derived from correlative bioinformatic analyses. The epigenetic regulation of gene expression and the tumor immune microenvironment is a multifactorial and dynamic process involving a network of interactions that cannot be fully captured by computational methods alone. Therefore, to validate the biological relevance of our findings, future research will focus on performing functional experiments, including gene knockdown/overexpression, CRISPR-based epigenetic editing, and immune cell co-culture systems. In particular, we plan to utilize *in vivo* melanoma models to investigate how *MYO1G* methylation status affects tumor progression and immune response in a physiological context. These follow-up studies will provide critical mechanistic insights and strengthen the translational significance of our findings.

## Conclusions

In conclusion, we first introduce the notion that *MYO1G* gene expression is regulated by promoter DNA methylation in melanoma. The associations of *MYO1G* gene expression and promoter hypomethylation with immune cell infiltrations and immune-related molecules reflect the impacts of *MYO1G* on anti-tumor immune response. Robust correlations of *MYO1G* gene expression and promoter hypomethylation with clinicopathological features and anti-tumor immune response firstly demonstrate that *MYO1G* gene expression and promoter hypomethylation are potential predictive biomarkers for immune cell infiltration, prognosis and immunotherapy response in melanoma. Our study also provides new insight for understanding the biological significance of *MYO1G* expression and promoter methylation in lymphocytes infiltration into tumor, which may help turn immune-cold tumor to hot and enhancing immunotherapy response.

## Data Availability

The datasets presented in this study can be found in online repositories. The names of the repository/repositories and accession number(s) can be found below: https://zenodo.org/records/15537847.

## Supplementary material

Supplementary Figure 1 Correlations of the methylation level of CpG sites with *MYO1G* gene expression. X-axis corresponds to Beta value of CpG site; Y-axis to log2-transformed gene expression value of *MYO1G.*
